# The Decrease in Estimated Glomerular Filtration Rate as a Risk Factor of Ventricular Tachyarrhythmias after Acute Myocardial Infarction during Hospitalization: A Retrospective Propensity Score Matching Cohort Study

**DOI:** 10.31083/j.rcm2502042

**Published:** 2024-01-29

**Authors:** Ming Li, Chongzhou Zheng, Chunmei Chen, Xifeng Zheng, Zhongkai He

**Affiliations:** ^1^Department of Cardiology, Affiliated Hospital of Guangdong Medical University, 524001 Zhanjiang, Guangdong, China

**Keywords:** estimated glomerular filtration rate, ventricular tachycardia, acute myocardial infarction, propensity score matching

## Abstract

**Background:**

To identify the decrease in estimated glomerular filtration rate (eGFR) as an independent risk factor associated with ventricular tachyarrhythmias (VTA).

**Methods:**

This retrospective file review collected information from patients diagnosed with acute myocardial infarction (AMI), with and without VTA, from January 2017 to December 2019. We first applied the chi-square test to assess 12 risk factors and one outcome variable (incident rate of VTA). Next, all the 12 risk factors were further adjusted using the propensity score matching (PSM) method to simulate the dataset as a randomized controlled cohort, which can reduce the defects derived from confounding factors and the imbalance in baseline characteristics. To investigate the relationship between eGFR and VTA, univariate logistic regression analysis was applied to the cohort before and after PSM analysis.

**Results:**

A total of 503 patients diagnosed as AMI were included in the study. There were eight of twelve risk factors in baseline characteristics with a *p*-value < 0.05, as determined by the chi-square test before PSM matching. The result of PSM analysis indicated that 86 of 91 patients with decreased eGFR were matched, and all the risk factors were not significantly different (*p*-value > 0.05). The incident rates of VTA in the two groups were still significantly different (*p*-value < 0.001) according to the Pearson chi-square test in the cohort after PSM analysis. The results of univariate (eGFR) logistic regression indicated that the odds ratio of the cohort was 6.442 (95% confidence interval = 3.770–11.05) and 3.654 (95% confidence interval = 1.764–7.993) before and after PSM analysis respectively.

**Conclusions:**

The decrease in eGFR (<60 mL/min/1.73 $m^{2}$) has been demonstrated as an independent risk factor for VTA after AMI.

## 1. Introduction

Ventricular tachyarrhythmias (VTA), encompassing ventricular tachycardia (VT), ventricular flutter, and ventricular fibrillation (VF), represents life-threatening complications that frequently manifest in the aftermath of acute myocardial infarction (AMI). The occurrence of VTA brings about a significant medical challenge, given the association with a substantial proportion of sudden cardiac deaths (SCD). In-hospital mortality approaches 20 percent or more in patients who accompany with VTA following by AMI [1, 2]. Rapid identification and treatment of high-risk patients susceptible to VTA is paramount in clinical cardiology. The interplay between renal function and cardiac disease is a subject of intricate reciprocity which could be summarized as cardiorenal syndrome (CRS). It describes a spectrum of disorders involving both the heart and kidneys in which acute or chronic dysfunction in one organ may induce acute or chronic dysfunction in the other organ [3]. Impaired renal function is increasingly recognized as a risk factor for adverse cardiovascular events including SCD [4, 5]. In this context, the estimated glomerular filtration rate (eGFR) emerges as a pivotal biological marker. Beyond its conventional role as an indicator of renal health, eGFR assumes significance as a predictive factor for cardiovascular events.

This premise leads us to a critical research question: Dose decrease in eGFR is an independent risk factor of the occurrence of VTA in patients with AMI? A brief retrospective review of existing literature reveals Anna C van der Burgh demonstrated that every 10 mL/min/1.73 $m^{2}$ eGFR-cys decrease was associated with 23% increase in the prevalence of SCD (HR = 1.23, with 95% confidence interval (CI) as 1.12–1.34, *p* < 0.001 [6]). However, due to interference from multiple risk factors, the specific investigation into the relationship between eGFR and the risk of VTA in patients with AMI remains inadequacy.

In this research, we employ the propensity score matching (PSM) method, which attempts to adjust post hoc for recognized unbalanced factors at baseline such that the data once analyzed will hopefully approximate or indicate what a prospective randomized dataset. It is well-regarded for its capacity to enhance the robustness of observational studies, allowing for an ideal assessment of correlation of interested variables in situations where randomized controlled trials may not be feasible [7]. Thus, the study aims to systematically explore the association between eGFR and VTA in the context of AMI rigorously, and provide more supporting evidence to the prevention of SCD.

## 2. Materials and Methods

### 2.1 The Study Design and Definition of the Risk Factors

The research was a retrospective cohort study and approved by the ethics committee of the Affiliated Hospital of Guangdong Medical University. Since to the retrospective nature of the analysis, the need for informed consent was waived.

Our team reviewed the information of hospitalized patients in electronic medical system that the primary discharge diagnosis as AMI, fulfilling the fourth universal definition of myocardial infarction (2018) [8] as inclusion criteria, from January 2017 to December 2019. We focus on the incident rate of VTA among the AMI patients. In this study, the diagnosis of VTA was validated through electrocardiogram (ECG) monitoring, Holter monitoring, or medical rescue treatment records. VTA refers to ventricular fibrillation, ventricular flutter, and sustained and non-sustained ventricular tachycardia, regardless of the presence of hemodynamic disorder. Thus, the symptoms of the patients suffering from VTA were heterogeneous, ranging from asymptomatic to sudden cardiac death, even with positive rescue treatment. Meanwhile, there were 13 kinds of variables were record, following as Age, Sex, Diabetes history, Hypertension history, Type of AMI, Number of diseased vessels, High-sensitivity troponin T (hs-TNT), N-terminal prohormone of brain natriuretic peptide (NT-proBNP), Percutaneous coronary intervention (PCI) treatment timing, Left ventricular ejection fraction, Hypokalemia, Infection during hospitalization and eGFR. The definitions and specific description of these 13 variables were listed in Table 1 (Ref. [1, 9, 10]). The case with incomplete clinical information of the 13 variables would be excluded. Given the retrospective design, our approach involved the comprehensive inclusion of clinical samples based on strict adherence to the inclusion and exclusion criteria. The cohort was divided into two groups base on the value of eGFR (eGFR <60 mL/min/1.73 $m^{2}$ or not), aims to identify the association between decrease in eGFR and VTA.

**Table 1.** **The risk factors with a definition in this study**.

Variables	Definition
1. Age	<60 years, 60–75 years, >75 years
2. Sex	Female/Male
3. Diabetes history	Yes/No
4. Hypertension history	Yes/No
5. Type of AMI	ST elevated myocardial infarction (STEMI)
	Non-ST elevated myocardial infarction (NSTEMI)
6. Number of diseased vessels	Single vessel/Double vessels/Triple vessels
7. hs-TNT	Less than 5 times the threshold (<0.50 ng/mL)
	More than 5 times the threshold (0.50 ng/mL–1.00 ng/mL)
	More than 10 times the threshold (>1.00 ng/mL)
8. NT-proBNP	Normal threshold
	Less than 5 times the threshold
	More than 5 times the threshold
9. PCI treatment timing	Without PCI treatment in hospitalized
	PCI treatment timing ≥24 h
	PCI treatment timing in 24 h
10. Left ventricular ejection fraction	>50%, 40%–50%, <40%
11. Hypokalemia	Yes (<3.5 mmol/L)/No (≥3.5 mmol/L)
12. Infection during hospitalization	Yes/No
13. eGFR	≥60 mL/min/1.73 $m^{2}$
	<60 mL/min/1.73 $m^{2}$

hs-TNT, high-sensitivity troponin T; AMI, acute myocardial infarction; NT-proBNP, N-terminal prohormone of brain natriuretic peptide; PCI, percutaneous coronary intervention; eGFR, estimated glomerular filtration rate. Note: Risk factors definition interpretation. 1. Number of diseased vessels: The number of diseased vessels was determined based on coronary angiography findings in three main cardiac vessels: the left main artery to the left anterior descending branch, the left circumflex artery, and the right coronary artery. A single lesion vessel was characterized by a main vessel or one of its branches with more than 75% stenosis. Meanwhile, double-lesion vessels were identified when a main vessel, along with another main vessel and/or its branches, showed more than 75% stenosis. The presence of more than 75% stenosis in all three main vessels and/or their branches classified them as triple-lesion vessels [1, 9]. 2. hs-TNT and NT-proBNP: The reference intervals of hsTnT were as: normal <0.01 ng/mL, myocardial injury: 0.01–0.1 ng/mL, considering myocardial infarction >0.10 ng/mL. There were also three reference intervals of NT-proBNP: age <50 years: less than 450 pg/mL, age 50–75 years: less than 900 pg/mL, age >75 years: less than 1800 pg/mL, respectively. The maximal values of hs-TNT and NT-proBNP for each patient during hospitalization were recorded and transformed into corresponding classified variables. 3. PCI treatment timing: The timing of PCI was calculated from the admission of the patient to the completion of the PCI procedure, as documented in the operation record. 4. Left ventricular ejection fraction (LVEF): The intervals of LVEF (>50%, 40%–50%, <40%) detected by echocardiography were followed the 2022 American College of Cardiology and American Heart Association guidelines for the management of heart failure [10]. 5. Infections during hospitalization, such as catheter-related infections or pneumonia, were defined based on whether they were accompanied by fever symptoms and/or antibiotic therapy. 6. eGFR: There were five intervals of eGFR: 90–120 mL/min/1.73 $m^{2}$, 60–89 mL/min/1.73 $m^{2}$, 30–59 mL/min/1.73 $m^{2}$, 15–29 mL/min/1.73 $m^{2}$, and <15 mL/min/1.73 $m^{2}$ respectively. The minimal eGFR value of each patient during hospitalization were recorded and transformed into corresponding classified variables.

### 2.2 Statistical Analysis

**Fig. 1.**
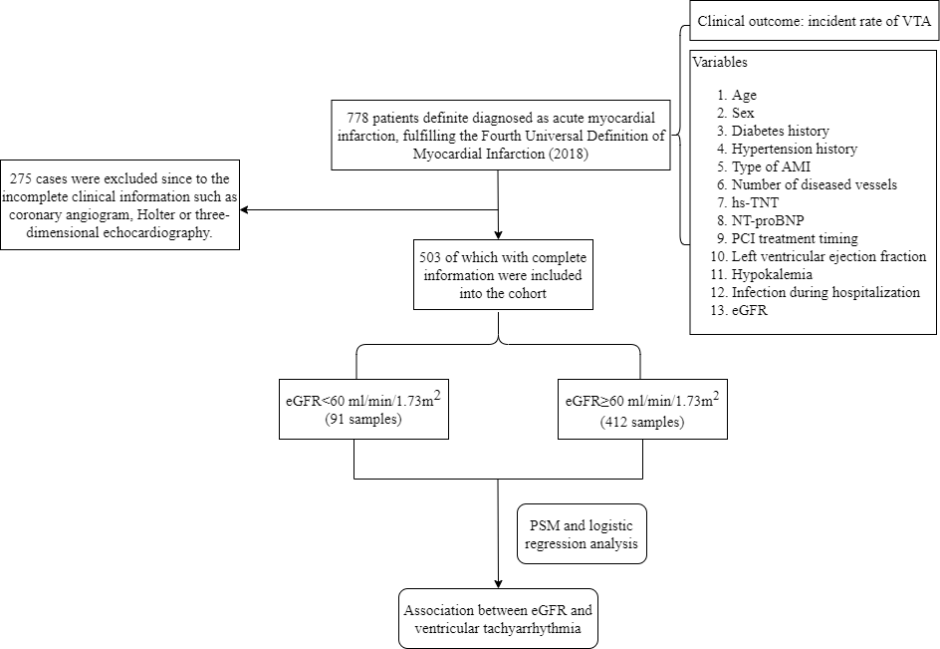
**The flowchart of the study**. hs-TNT, high-sensitivity troponin T; AMI, acute myocardial infarction; NT-proBNP, N-terminal prohormone of brain natriuretic peptide; PCI, percutaneous coronary intervention; VTA, ventricular tachyarrhythmias; eGFR, estimated glomerular filtration rate; PSM, propensity score matching.

The 12 variables (Age, Sex, Diabetes history, Hypertension history, Type of AMI, Number of diseased vessels, hs-TNT, NT-proBNP, PCI treatment timing, Left ventricular ejection fraction, Hypokalemia, Infection during hospitalization) and one outcome variable (incident rate of VTA) were recorded as categorical variables and were expressed as numbers and percentages by the Chi-square test. The variables with *p*-value < 0.05 were regarded as statistically significant. Accounting for the difference in baseline characteristics of the two groups, the PSM method, which was estimated by multivariable logistic-regression model, was applied to adjust all 12 variables, to eliminate the effect of confounding factors [11]. The process was performed with a ratio of 1:1 matching protocol by optimal matching arithmetic. Univariate (eGFR) logistic regression analysis was applied to develop a model with the cohort before and after PSM analysis, respectively, to determine the association between eGFR and VTA. The information on eGFR was expressed as odds ratios (OR) with 95% CI and *p*-values. The receiver operating characteristic (ROC) curves were used to illustrate the discrimination ability. Statistical analysis was performed using R software (version 4.0.3, Foundation for Statistical Computing, Vienna, Austria) and “MatchIt” and “pROC” packages. The flowchart of the study was illustrated as Fig. 1.

## 3. Results

### 3.1 The Baseline Information and Results of PSM Analysis

**Fig. 2.**
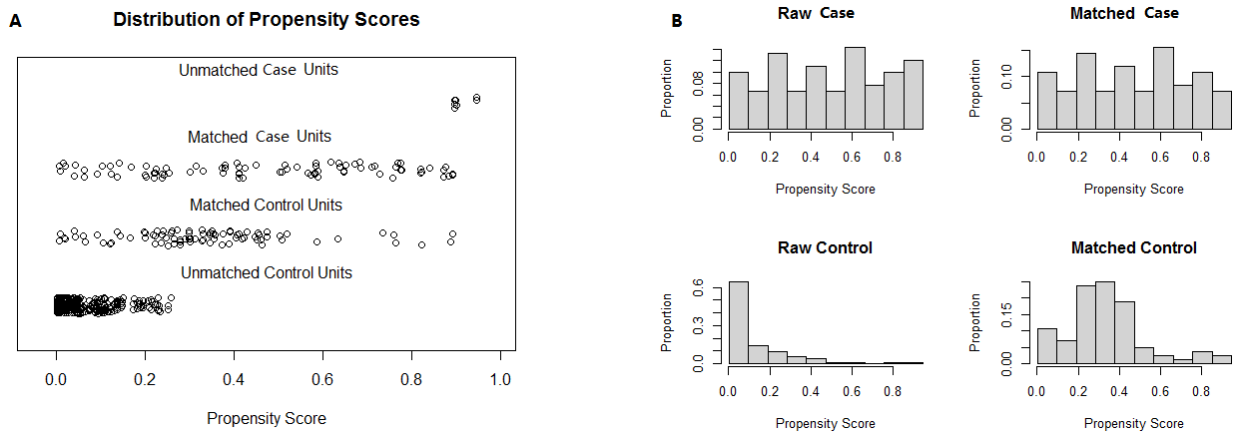
**The result of PSM analysis illustrated by scatter plot and histogram**. Note: (A) is a scatter plot, while (B) is a histogram. The case and control samples were imbalanced in the raw dataset and were adjusted by PSM analysis. PSM, propensity score matching.

According to the statistic, there were totally 778 patients clearly diagnosis as AMI during the period. However, 275 cases of which were excluded since to the incomplete clinical information primarily centered on coronary angiogram, Holter or three-dimensional echocardiography. Among the 503 samples including in this study cohort, 91 had suffered kidney damage with a decrease in eGFR below 60 mL/min/1.73 $m^{2}$, accounting for 18.1% of the total samples. There were eight variables (age, number of diseased vessels, PCI treatment timing, diabetes history, infection during hospitalization, hsTnT, NT-proBNP, and left ventricular ejection fraction (LVEF)) with a *p*-value < 0.05, according to the chi-square test. The PSM method was applied to adjust the 12 variables, and 86 patients out of 91 were matched (Fig. 2). The new dataset with 172 samples after PSM analysis could be considered a cohort that eliminates the effects of the known confounding factors (Table 2).

**Table 2.** **The cohort before and after PSM analysis**.

Variables		Before PSM analysis				After PSM analysis			
		Total (n = 503)	eGFR <${\mathrm{60}}^{a}$ (n = 91)	eGFR ≥${\mathrm{60}}^{a}$ (n = 412)	*p*-value	Total (n = 172)	eGFR <${\mathrm{60}}^{a}$ (n = 86)	eGFR ≥${\mathrm{60}}^{a}$ (n = 86)	*p*-value
Age, n (%)					<0.001*				0.821
	<60 y	71 (14.11)	5 (5.49)	66 (16.02)		12 (6.98)	5 (5.82)	7 (8.14)	
	60 y–75 y	244 (48.51)	35 (38.46)	209 (50.73)		66 (38.37)	34 (39.53)	32 (37.21)	
	>75 y	188 (37.38)	51 (56.05)	137 (33.25)		94 (54.65)	47 (54.65)	47 (54.65)	
Sex, n (%)					0.745				0.871
	Female	176 (35.00)	30 (32.97)	146 (35.44)		56 (32.56)	27 (31.40)	29 (33.72)	
	Male	327 (65.00)	61 (67.03)	266 (64.56)		116 (67.44)	59 (68.60)	57 (66.28)	
STEMI, n (%)					1.000				1.000
	No	265 (52.68)	48 (52.75)	217 (52.67)		86 (50.00)	43 (50.00)	43 (50.00)	
	Yes	238 (47.32)	43 (47.25)	195 (47.33)		86 (50.00)	43 (50.00)	43 (50.00)	
Number of diseased vessels, n (%)					<0.001*				0.391
	Single vessel	250 (49.70)	12 (13.19)	238 (57.77)		29 (16.86)	12 (13.95)	17 (19.77)	
	Double vessels	149 (29.62)	34 (37.36)	115 (27.91)		71 (41.28)	34 (39.54)	37 (43.02)	
	Triple vessels	104 (20.68)	45 (49.45)	59 (14.32)		72 (41.86)	40 (46.51)	32 (37.21)	
PCI treatment timing, n (%)					<0.001*				0.356
	No PCI treatment	85 (16.90)	35 (38.46)	50 (12.13)		52 (30.23)	30 (34.88)	22 (25.58)	
	PCI treatment timing ≥24 h	192 (38.17)	20 (21.98)	172 (41.75)		46 (26.74)	20 (23.26)	26 (30.23)	
	PCI treatment timing in 24 h	226 (44.93)	36 (39.56)	190 (46.12)		74 (43.03)	36 (41.86)	38 (44.19)	
Hypokalemia, n (%)					0.492				0.121
	No	470 (93.44)	87 (95.60)	383 (92.96)		168 (97.67)	82 (95.34)	86 (100)	
	Yes	33 (6.56)	4 (4.40)	29 (7.04)		4 (2.33)	4 (4.66)	0 (0)	
Diabetes history, n (%)					<0.001*				0.756
	No	387 (76.94)	50 (54.95)	337 (81.80)		103 (59.88)	50 (58.14)	53 (61.63)	
	Yes	116 (23.06)	41 (45.05)	75 (18.20)		69 (40.12)	36 (41.86)	33 (38.37)	
Hypertension history, n (%)					0.739				0.878
	No	298 (59.24)	52 (57.14)	246 (59.71)		98 (56.98)	50 (58.14)	48 (55.81)	
	Yes	205 (40.76)	39 (42.86)	166 (40.29)		74 (43.02)	36 (41.86)	38 (44.19)	
Infection, n (%)					<0.001*				0.081
	No	414 (82.31)	49 (53.85)	365 (88.59)		110 (63.95)	49 (56.98)	61 (70.93)	
	Yes	89 (17.69)	42 (46.15)	47 (11.41)		62 (36.05)	37 (43.02)	25 (29.07)	
NT-proBNP, n (%)					<0.001*				0.097
	Normal	137 (27.24)	9 (9.89)	128 (31.07)		25 (14.53)	9 (10.46)	16 (18.60)	
	Less than 5 times threshold	264 (52.49)	33 (36.26)	231 (56.07)		70 (40.70)	32 (37.21)	38 (44.19)	
	More than 5 times threshold	102 (20.27)	49 (53.85)	53 (12.86)		77 (44.77)	45 (52.33)	32 (37.21)	
LVEF, n (%)					<0.001*				0.144
	>50%	300 (59.64)	31 (34.07)	269 (65.29)		66 (38.37)	29 (33.72)	37 (43.02)	
	40%–50%	170 (33.80)	43 (47.25)	127 (30.83)		84 (48.84)	42 (48.84)	42 (48.84)	
	<40%	33 (6.56)	17 (18.68)	16 (3.88)		22 (12.79)	15 (17.44)	7 (8.14)	
hs-TnT, n (%)					<0.001*				0.373
	Less than 5 times threshold	109 (21.67)	15 (16.48)	94 (22.82)		35 (20.35)	15 (17.44)	20 (23.26)	
	More than 5 times threshold	168 (33.40)	15 (16.48)	153 (37.13)		34 (19.77)	15 (17.44)	19 (22.09)	
	More than 10 times threshold	226 (44.93)	61 (67.04)	165 (40.05)		103 (59.88)	56 (65.12)	47 (54.65)	
Outcome status, n (%)					<0.001*				<0.001*
	Non-VTA	429 (85.29)	55 (60.44)	374 (90.78)		128 (74.42)	54 (62.79)	74 (86.05)	
	VTA	74 (14.71)	36 (39.56)	38 (9.22)		44 (25.58)	32 (37.21)	12 (13.95)	

hs-TnT, high-sensitivity troponin T; NT-proBNP, N-terminal prohormone of brain natriuretic peptide; PCI, percutaneous coronary intervention; VTA, ventricular tachyarrhythmias; LVEF, left ventricular ejection fraction; eGFR, estimated glomerular filtration rate; PSM, propensity score matching; STEMI, ST elevated myocardial infarction. Note: ^a^, mL/min/1.73 $m^{2}$. “*” means the variable with statistically significance.

### 3.2 The Result of Univariate (eGFR) Logistic Regression Analysis

**Fig. 3.**
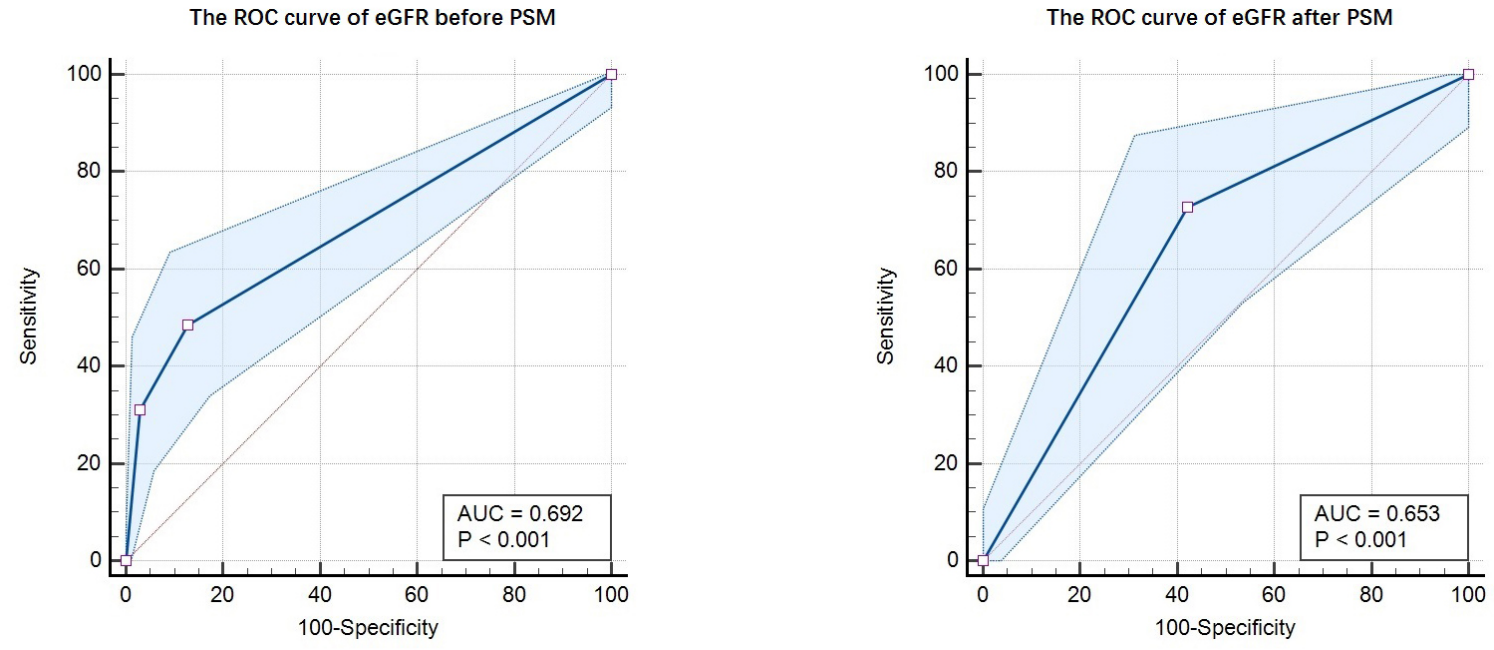
**The ROC curve of eGFR before and after PSM**. Note: The AUC of ROC curve before PSM was 0.692 (95% CI = 0.649–0.732), with sensitivity as 48.65% (95% CI = 36.9%–60.6%), specificity as 87.18% (95% CI = 83.6%–90.2%). The AUC of ROC curve after PSM was 0.653 (95% CI = 0.576–0.724), with sensitivity as 72.73% (95% CI = 57.2%–85%), specificity as 57.81% (95% CI = 48.8%–66.5%). ROC, receiver operating characteristic; eGFR, estimated glomerular filtration rate; AUC, area under the curve; PSM, propensity score matching.

Univariate (eGFR) logistic regression analysis was performed to develop a model based on the original cohort and the cohort after PSM. The OR of the cohort were 6.442 (95% CI = 3.770–11.05), and 3.654 (95% CI = 1.764–7.993) before and after PSM analysis, respectively (Table 3). Meanwhile, the ROC curve of eGFR in the cohort before and after PSM were illustrated as Fig. 3. The area under the curve (AUC) of ROC curve before PSM was 0.692 (95% CI = 0.649–0.732), with sensitivity as 48.65% (95% CI = 36.9%–60.6%), specificity as 87.18% (95% CI = 83.6%–90.2%). The AUC of ROC curve after PSM was 0.653 (95% CI = 0.576–0.724), with sensitivity as 72.73% (95% CI = 57.2%–85%), specificity as 57.81% (95% CI = 48.8%–66.5%). The result proved that a decrease in eGFR (<60 mL/min/1.73 $m^{2}$) is an independent risk factor associated with VTA, in patients with acute myocardial infarction.

**Table 3.** **The result of univariate (eGFR) logistic regression analysis**.

Variable		β	Odds ratio (95% CI)	*p*-value
Before PSM analysis				
	eGFR (≥60 mL/min/1.73 $m^{2}$)	–2.287	0.102 (0.072–0.140)	<0.001
	eGFR (<60 mL/min/1.73 $m^{2}$)	1.863	6.442 (3.770–11.05)	<0.001
After PSM analysis				
	eGFR (≥60 mL/min/1.73 $m^{2}$)	–1.819	0.162 (0.084–0.287)	<0.001
	eGFR (<60 mL/min/1.73 $m^{2}$)	1.296	3.654 (1.764–7.993)	<0.001

Note: β, regression coefficient; CI, confidence interval; eGFR, estimated glomerular filtration rate; PSM, propensity score matching.

## 4. Discussion

The definition and classification of chronic kidney disease (CKD) based on glomerular filtration rate (GFR), raised in 2002 and acknowledged since 2004, has been followed until today [12, 13]. eGFR is a common indicator based on serum creatinine levels, age, sex and cystatin C (according to the 2012 Chronic Kidney Disease Epidemiology Collaboration (CKD-EPI) cystatin C equation), which is ideal for reflecting kidney function and is considered an alternative to GFR in clinical practice [14]. The relationship between kidney damage and major adverse cardiovascular events remains a concern for researchers. In this study, we aimed to explore the relationship between the decrease in eGFR and the incidence rate of VTA in AMI patients. The PSM method was applied to simulate the cohort as a randomized controlled trial, to reduce the effects of different confounding factors. The result of PSM analysis showed that 86 of 91 patients with renal dysfunction were matched. The univariate (eGFR) logistic regression analysis demonstrated that the OR of the original cohort was 6.442 (95% CI = 3.770–11.05), and the OR of the cohort after PSM analysis was 3.654 (95% CI = 1.764–7.993). The study concluded that the decrease in eGFR (<60 mL/min/1.73 $m^{2}$) is a risk factor associated with the concurrence of VTA in patients with AMI.

According to previous studies, CKD is considered to be one of the strongest risk factors for the development of cardiovascular disease [15]. The prevalence of cardiovascular disease among patients older than 65 years of age with CKD in the United States is 64.5%, compared with only 32.4% among those without CKD [16]. Several meta-analyses with large samples have shown that a decrease in eGFR (<60 mL/min/1.73 $m^{2}$) was significantly associated with an increased risk of all-cause and cardiovascular mortality, regardless of the high-risk population or the general population cohort, or independent of traditional risk factors (such as hypertension, diabetes, and hyperlipidemia) [17, 18]. According to the 2018 U.S. Renal Data System data, non-atherosclerotic adverse cardiovascular events, such as SCD or fatal arrhythmias, are more common in patients with end-stage renal disease (ESRD) than in those with atherosclerosis-related complications, such as AMI or stroke. The report noted that up to 40% of ESRD patients died of SCD, while 18% died of acute myocardial infarction [16]. A recent study highlighted a 6 to 20-fold increased risk of SCD in patients exposed to chronic kidney failure compared with the non-exposed population [19]. Our previous study suggested that eGFR <60 mL/min/1.73 $m^{2}$, especially <30 mL/min/1.73 $m^{2}$ is a significant variable in the prediction model of SCD after AMI [20]. This opinion is supported by both Faxén *et al*. [21] and Docherty *et al*. [22]. All this clinical evidence confirmed that the decrease in eGFR was an independent risk factor for ventricular tachyarrhythmias after AMI.

Cardiac structural remodeling and electrophysiological changes in patients with CKD are the main causes of fatal arrhythmias. Sympathetic hyperactivity is evident at the earliest stage of CKD, which can trigger adratic-related ventricular tachyarrhythmias in susceptible individuals and is directly related to the progression of renal failure [23, 24]. Several studies have demonstrated that renal denervation can decrease the susceptibility of the heart to ventricular fibrillation, in dog and rabbit CKD models [25, 26]. Cardiac structural remodeling includes left ventricular hypertrophy (LVH) and myocardial fibrosis. LVH is easily detected on electrocardiography or echocardiography. The Framingham study followed more than 3000 samples over 14 years and concluded that SCD in the normal population was 1.64%, while SCD in the LVH population was up to 21.5% [27]. Overactivity of the renin–angiotensin–aldosterone system (RAAS) is the most important mechanism of myocardial hypertrophy as it can promote vasoconstriction, cardiac ischemia, myocardial apoptosis, and fibrosis, which are fundamental to ventricular tachyarrhythmias [28]. Myocardial fibrosis is a common pathological state in various cardiac diseases and is an essential cause of ventricular tachyarrhythmia caused by reentrant activity and initiation trigger mechanisms. Cardiac magnetic resonance imaging can directly reflect the degree of myocardial fibrosis through late gadolinium enhancement (LGE) [29]. Multiple clinical studies and meta-analyses have shown that ventricular fibrosis detected using LGE is a powerful predictor of SCD events in patients with ischemic heart disease, dilated cardiomyopathy, and hypertrophic cardiomyopathy [30, 31, 32]. Moreover, ventricular arrhythmias and SCD in patients with ESRD may be related to sharp changes in blood pressure, hypovolemia, and electrolyte disturbance caused by dialysis treatment [33, 34].

Nevertheless, some limitations need to be mentioned. First, this was a single-center retrospective study and the selective bias was inevitable. Moreover, the risk factors related to VTA were numerous, while the study did not cover certain aspects, such as body mass index, drug treatment, the location of the culprit’s vessel, and history of heart failure.

## 5. Conclusions

The result of our study has demonstrated the decrease in eGFR (<60 mL/min/1.73 $m^{2}$) as an independent risk factor of VTA after AMI by PSM analysis.

## Data Availability

The datasets used and/or analyzed during the current study are available from the corresponding author on reasonable request.

## Abbreviations

eGFR, estimated glomerular filtration rate; VTA, ventricular tachyarrhythmia; AMI, acute myocardial infarction; PSM, propensity score matching; OR, odds ratio; CI, confidence interval; LVEF, left ventricular ejection fraction; ESRD, end-stage renal disease; CKD, chronic kidney disease.

## References

[1] Al-Khatib SM, Stevenson WG, Ackerman MJ, Bryant WJ, Callans DJ, Curtis AB (2018). 2017 AHA/ACC/HRS guideline for management of patients with ventricular arrhythmias and the prevention of sudden cardiac death: A Report of the American College of Cardiology/American Heart Association Task Force on Clinical Practice Guidelines and the Heart Rhythm Society. *Heart Rhythm*.

[2] Jong-Ming Pang B, Green MS (2017). Epidemiology of ventricular tachyarrhythmia: Any changes in the past decades. *Herzschrittmachertherapie & Elektrophysiologie*.

[3] Rangaswami J, Bhalla V, Blair JEA, Chang TI, Costa S, Lentine KL (2019). Cardiorenal Syndrome: Classification, Pathophysiology, Diagnosis, and Treatment Strategies: A Scientific Statement From the American Heart Association. *Circulation*.

[4] Ganesha Babu G, Webber M, Providencia R, Kumar S, Gopalamurugan A, Rogers DP (2016). Ventricular Arrhythmia Burden in Patients With Heart Failure and Cardiac Resynchronization Devices: The Importance of Renal Function. *Journal of Cardiovascular Electrophysiology*.

[5] Vazquez R, Bayes-Genis A, Cygankiewicz I, Pascual-Figal D, Grigorian-Shamagian L, Pavon R (2009). The MUSIC Risk score: a simple method for predicting mortality in ambulatory patients with chronic heart failure. *European Heart Journal*.

[6] van der Burgh AC, Stricker BH, Rizopoulos D, Ikram MA, Hoorn EJ, Chaker L (2022). Kidney function and the risk of sudden cardiac death in the general population. *Clinical Kidney Journal*.

[7] Haukoos JS, Lewis RJ (2015). The Propensity Score. *JAMA*.

[8] Thygesen K, Alpert JS, Jaffe AS, Chaitman BR, Bax JJ, Morrow DA (2018). Fourth Universal Definition of Myocardial Infarction (2018). *Circulation*.

[9] Oe K, Shimizu M, Ino H, Yamaguchi M, Terai H, Hayashi K (2002). Effects of gender on the number of diseased vessels and clinical outcome in Japanese patients with acute coronary syndrome. *Circulation Journal: Official Journal of the Japanese Circulation Society*.

[10] Heidenreich PA, Bozkurt B, Aguilar D, Allen LA, Byun JJ, Colvin MM (2022). 2022 AHA/ACC/HFSA Guideline for the Management of Heart Failure: A Report of the American College of Cardiology/American Heart Association Joint Committee on Clinical Practice Guidelines. *Circulation*.

[11] Ahmed A, Husain A, Love TE, Gambassi G, Dell’Italia LJ, Francis GS (2006). Heart failure, chronic diuretic use, and increase in mortality and hospitalization: an observational study using propensity score methods. *European Heart Journal*.

[12] National Kidney Foundation (2002). K/DOQI clinical practice guidelines for chronic kidney disease: evaluation, classification, and stratification. *American Journal of Kidney Diseases: the Official Journal of the National Kidney Foundation*.

[13] Levey AS, Eckardt KU, Tsukamoto Y, Levin A, Coresh J, Rossert J (2005). Definition and classification of chronic kidney disease: a position statement from Kidney Disease: Improving Global Outcomes (KDIGO). *Kidney International*.

[14] Levey AS, Inker LA, Coresh J (2014). GFR estimation: from physiology to public health. *American Journal of Kidney Diseases: the Official Journal of the National Kidney Foundation*.

[15] Herzog CA, Asinger RW, Berger AK, Charytan DM, Díez J, Hart RG (2011). Cardiovascular disease in chronic kidney disease. A clinical update from Kidney Disease: Improving Global Outcomes (KDIGO). *Kidney International*.

[16] Saran R, Robinson B, Abbott KC, Agodoa LYC, Bragg-Gresham J, Balkrishnan R (2019). US Renal Data System 2018 Annual Data Report: Epidemiology of Kidney Disease in the United States. *American Journal of Kidney Diseases: the Official Journal of the National Kidney Foundation*.

[17] Matsushita K, van der Velde M, Astor BC, Woodward M, Levey AS, Chronic Kidney Disease Prognosis Consortium (2010). Association of estimated glomerular filtration rate and albuminuria with all-cause and cardiovascular mortality in general population cohorts: a collaborative meta-analysis. *The Lancet (London, England)*.

[18] van der Velde M, Matsushita K, Coresh J, Astor BC, Woodward M, Levey A (2011). Lower estimated glomerular filtration rate and higher albuminuria are associated with all-cause and cardiovascular mortality. A collaborative meta-analysis of high-risk population cohorts. *Kidney International*.

[19] Svane J, Nielsen JL, Stampe NK, Feldt-Rasmussen B, Garcia R, Risgaard B (2022). Nationwide study of mortality and sudden cardiac death in young persons diagnosed with chronic kidney disease. *Europace: European Pacing, Arrhythmias, and Cardiac Electrophysiology: Journal of the Working Groups on Cardiac Pacing, Arrhythmias, and Cardiac Cellular Electrophysiology of the European Society of Cardiology*.

[20] Zheng X, Huang R, Liu G, Jia Z, Chen K, He Y (2021). Development and verification of a predictive nomogram to evaluate the risk of complicating ventricular tachyarrhythmia after acute myocardial infarction during hospitalization: A retrospective analysis. *The American Journal of Emergency Medicine*.

[21] Faxén J, Jernberg T, Hollenberg J, Gadler F, Herlitz J, Szummer K (2020). Incidence and Predictors of Out-of-Hospital Cardiac Arrest Within 90 Days After Myocardial Infarction. *Journal of the American College of Cardiology*.

[22] Docherty KF, Ferreira JP, Sharma A, Girerd N, Gregson J, Duarte K (2020). Predictors of sudden cardiac death in high-risk patients following a myocardial infarction. *European Journal of Heart Failure*.

[23] Manolis AA, Manolis TA, Apostolopoulos EJ, Apostolaki NE, Melita H, Manolis AS (2021). The role of the autonomic nervous system in cardiac arrhythmias: The neuro-cardiac axis, more foe than friend. *Trends in Cardiovascular Medicine*.

[24] Kiuchi MG, Ho JK, Nolde JM, Gavidia LML, Carnagarin R, Matthews VB (2020). Sympathetic Activation in Hypertensive Chronic Kidney Disease - A Stimulus for Cardiac Arrhythmias and Sudden Cardiac Death. *Frontiers in Physiology*.

[25] Tang X, Shi L, Cui X, Yu Y, Qi T, Chen C (2017). Renal denervation decreases susceptibility of the heart to ventricular fibrillation in a canine model of chronic kidney disease. *Experimental Physiology*.

[26] Liu SH, Lo LW, Chou YH, Lin WL, Tsai TY, Cheng WH (2021). Renal denervation prevents myocardial structural remodeling and arrhythmogenicity in a chronic kidney disease rabbit model. *Heart Rhythm*.

[27] Haider AW, Larson MG, Benjamin EJ, Levy D (1998). Increased left ventricular mass and hypertrophy are associated with increased risk for sudden death. *Journal of the American College of Cardiology*.

[28] Giamouzis G, Dimos A, Xanthopoulos A, Skoularigis J, Triposkiadis F (2022). Left ventricular hypertrophy and sudden cardiac death. *Heart Failure Reviews*.

[29] Disertori M, Masè M, Ravelli F (2017). Myocardial fibrosis predicts ventricular tachyarrhythmias. *Trends in Cardiovascular Medicine*.

[30] Weng Z, Yao J, Chan RH, He J, Yang X, Zhou Y (2016). Prognostic Value of LGE-CMR in HCM: A Meta-Analysis. *JACC. Cardiovascular Imaging*.

[31] De Angelis G, De Luca A, Merlo M, Nucifora G, Rossi M, Stolfo D (2022). Prevalence and prognostic significance of ischemic late gadolinium enhancement pattern in non-ischemic dilated cardiomyopathy. *American Heart Journal*.

[32] Chery G, Kamp N, Kosinski AS, Schmidler GS, Lopes RD, Patel M (2020). Prognostic value of myocardial fibrosis on cardiac magnetic resonance imaging in patients with ischemic cardiomyopathy: A systematic review. *American Heart Journal*.

[33] Di Lullo L, Rivera R, Barbera V, Bellasi A, Cozzolino M, Russo D (2016). Sudden cardiac death and chronic kidney disease: From pathophysiology to treatment strategies. *International Journal of Cardiology*.

[34] Green D, Roberts PR, New DI, Kalra PA (2011). Sudden cardiac death in hemodialysis patients: an in-depth review. *American Journal of Kidney Diseases: the Official Journal of the National Kidney Foundation*.

